# IL-10^−/−^ Enhances DCs Immunity Against *Chlamydia psittaci* Infection *via* OX40L/NLRP3 and IDO/Treg Pathways

**DOI:** 10.3389/fimmu.2021.645653

**Published:** 2021-05-21

**Authors:** Qiang Li, Xiaohui Li, Hongkun Quan, Yihui Wang, Guanggang Qu, Zhiqiang Shen, Cheng He

**Affiliations:** ^1^Key Lab of Animal Epidemiology and Zoonoses of Ministry of Agriculture and Rural Affairs, College of Veterinary Medicine, China Agricultural University, Beijing, China; ^2^Preventive Veterinary Research Group, Binzhou Animal Science and Veterinary Medicine Academy of Shandong Province, Binzhou, China

**Keywords:** *Chlamydia psittaci*, interleukin-10, dendritic cells, OX40-OX40L, NLRP3 inflammasome, apoptosis

## Abstract

*Chlamydia psittaci* (*C. psittaci*) is a common zoonotic agent that affects both poultry and humans. Interleukin 10 (IL-10) is an anti-inflammatory factor produced during chlamydial infection, while dendritic cells (DCs) are powerful antigen-presenting cells that induce a primary immune response in the host. However, IL-10 and DCs regulatory mechanisms in *C. psittaci* infection remain elusive. *In vivo* and *in vitro* investigations of the regulatory mechanisms were performed. IL-10^−/−^ mice, conditional DCs depletion mice (zinc finger dendritic cell-diphtheria toxin receptor [zDC-DTR]), and double-deficient mice (DD, IL-10^−/−^/zDC^DTR/DTR^) were intranasally infected with *C. psittaci*. The results showed that more than 90% of IL-10^−/−^ mice, 70% of wild-type mice, and 60% of double-deficient mice survived, whereas all zDC-DTR mice died. A higher lymphocyte proliferation index was found in the IL-10 inhibitor mice and IL-10^−/−^ mice. Moreover, severe lesions and high bacterial loads were detected in the zDC-DTR mice compared with double-deficient mice. *In vitro* studies revealed increased OX40-OX40 ligand (OX40-OX40L) activation and CD4^+^T cell proliferation. Besides, the expression of indoleamine 2, 3-dioxygenase (IDO), and regulatory T cells were significantly reduced in the co-culture system of CD4^+^ T cells and IL-10^−/−^ DCs in *C. psittaci* infection. Additionally, the activation of the NLR family pyrin domain-containing 3 (NLRP3) inflammasome increased to facilitate the apoptosis of DCs, leading to rapid clearance of *C. psittaci*. Our study showed that IL-10^−/−^ upregulated the function of deficient DCs by activating OX40-OX40L, T cells, and the NLPR3 inflammasome, and inhibiting IDO, and regulatory T cells. These effects enhanced the survival rate of mice and *C. psittaci* clearance. Our research highlights the mechanism of IL-10 interaction with DCs, OX40-OX40L, and the NLPR3 inflammasome, as potential targets against *C. psittaci* infection.

## Introduction

*Chlamydia psittaci* (*C. psittaci*) is an obligate intracellular Gram-negative pathogen and an important zoonotic agent. *C. psittaci* infection causes severe avian respiratory diseases and human atypical pneumonia, leading to considerable economic losses and it is a potential threat to human health (1). Virulent *C. psittaci* exacerbates host immunosuppression and enhances secondary avian influenza virus serotype H9N2 infection by impairing macrophage functions and upregulating T helper 2 (Th2) cytokines, as well as the expression of interleukin 4 (IL-4) and IL-10 (2, 3).

IL-10 is firstly identified as a Th1 immune response inhibitory factor. It is secreted by T cells and antigen-presenting cells (APCs). Although IL-10 can stimulate the activity of B cells, natural killer cells, and mast cells, it is generally regarded as an immunosuppressive factor that promotes chronic bacterial and viral infections (4). IL-10 contributes to the progression of intracellular pathogens, including *Coxiella burnetii* (5), and *Mycobacterium tuberculosis* (6). Patients infected with *Chlamydia trachomatis* have shown increased IL-10 production in the cervix, and higher IL-10 levels have also been reported in infertile women (7). Recently, B cells are the main source of IL-10 following *Chlamydia muridarum* infections, and are reported to modulate the immune response by establishing chronic infections both *in vitro* and *in vivo* (8). IL-10 nanoparticle capsules as therapeutic strategies can modulate the release of IL-6, IL-12p40, suppressor of cytokine signaling 1 (SOCS1), and SOCS3 by mitigating the inflammatory response against Chlamydia infection in macrophages (9). Therefore, understanding the role of IL-10 is essential for the control of Chlamydia infection.

Although dendritic cells (DCs) play a significant role in anti-infection immune response, understanding the crosstalk between DCs and IL-10 is essential for elucidating the mechanism of immune escape during Chlamydia infection. Transforming growth factor-β1 (TGF-β1)-treated DCs resist the maturation stimulus of lipopolysaccharides by downregulating the expression of toll-like receptor 4 (TLR4) on DCs and increasing IL-10 expression (10). As an immunosuppressive cytokine, IL-10 is a crucial factor for the induction of immune-tolerant DCs through inhibition of Th1 cell antigen presentation and differentiation (11). For intracellular pathogens such as *Listeria monocytogenes*, DCs function is impaired *via* the IL-10-dependent crosstalk between marginal zone B cells and CD8α^+^ DCs (12). Additionally, IL-10 can regulate DCs to suppress specific Th2 immune responses (13). *Mycobacterium tuberculosis* mediates IL-10 production and alters the immune tolerance of DCs by inhibiting the Th1 response (14). Moreover, regulatory T cells (Tregs) are vital immune-regulatory cells that regulate immune homeostasis with effector T cells (15). Besides, Tregs also downregulates the expression of co-stimulatory molecules on the surface of APCs, blocks the activation of effector T cells, and enhances IL-10, IDO, and TGF-β expression (16). However, the association of Tregs with IL-10 and DCs during Chlamydia infection remains unknown.

Nod-like receptors (NLRs) identify pathogen invasion by activating innate immunity (17). The NLR family pyrin domain-containing 3 (NLRP3) inflammasome plays an important anti-infection role. *Chlamydia abortus* polymorphic membrane protein D (PmpD) 18.1 activates the NLRP3 inflammasome to induce TLR4/MyD88/nuclear factor-κB pathway-mediated secretion of IL-1β and contributes to the maturation and activation of DCs (18). The NLRP3 inflammasome activates DCs and mediates apoptosis to maintain DCs homeostasis against microbial infection (19). However, the immune response of the NLRP3 inflammasome and DCs apoptosis during *C. psittaci* infection remains unclear. Moreover, elucidating the mechanism by which the IL-10-mediated NLRP3 inflammasome functions is essential for understanding of Chlamydia infection.

In this study, we hypothesized that IL-10 deficiency may increase DCs function and Th1 immune response *via* recognition receptors and apoptosis pathways against *C. psittaci* infection. *In vivo* experiments were performed, and mortality, lesions, and bacterial loads were monitored following *C. psittaci* infection. DCs maturation, Tregs, OX40-OX40L receptor expression, and NLRP3 inflammasome function were detected in a co-culture system of CD4^+^T cells and DCs using primary bone marrow-derived dendritic cells (BMDCs) isolated from IL-10^−/−^ mice, conditional DCs depletion mice [zDC-diphtheria toxin receptor (zDC-DTR)], and double-deficient (DD) mice.

## Materials and Methods

### Animals and Ethics Statement

The protocols used in this study were approved by the Committee on Experimental Animal Management of China Agricultural University (Beijing, China). All animals were handled in strict accordance with the Regulations for the Administration of Affairs Concerning Experimental Animals of the State Council of the People’s Republic of China. Humane protocols that minimize pain to the animals were followed. In brief, mice were monitored daily for any clinical signs. Mice displaying severe depression were removed and euthanized. At the end of the study, all animals were euthanized using 100% CO_2_ at a flow rate of 50% of the chamber volume per minute. Death was confirmed by the absence of breathing and lack of a heartbeat, and additional secondary physical euthanasia (cervical dislocation) was performed before tissue collection and carcass disposal.

Eight-week-old female C57BL/6 mice, IL-10 knockout (002250) mice, and zDC-DTR (019506) mice were purchased from the Jackson Laboratory (Bar Harbor, ME, USA). DD mice (IL-10^−/−^/zDC^DTR/DTR^) were generated by breeding IL-10^−/−^ mice with zDC-DTR mice. All mouse genotypes were verified by polymerase chain reaction (PCR) according to the protocols provided by the Jackson Laboratory (**Supplemental Figure S1**). Mouse breeding was performed by a commercial company (Vitalstar Biotechnology Co., Ltd, Beijing, China). All mice were housed in specific pathogen-free (SPF) conditions and exposed to a 12-h light/dark cycle. During animal experiments, all efforts were made to minimize animal suffering.

### *C. psittaci* Strains

The *C. psittaci* 6BC strain was donated by Professor Yimou Wu from the University of Southern China, propagated by infecting Buffalo green monkey kidney cells, and incubated at 37°C in a 5% CO_2_ incubator for 72 h. Live elementary bodies (EBs) were harvested and purified by density gradient centrifugation, and the number of inclusion-forming units (IFUs) was determined before *in vivo* and *vitro* experiments using Chlamydia immunofluorescence assay (IMAGEN™; Oxoid, Cambridge, UK). Live EBs were stored in sucrose phosphate glutamate buffer at −70°C (20).

### Experimental Procedures

Before the experiment, to induce conditional DCs deletion, zDC-DTR mice, and DD mice were intraperitoneally injected with 20 ng of diphtheria toxin (DT) (D0564; Sigma–Aldrich, MI, USA) per gram of body weight. The mice were euthanized within 48 h to collect the bone marrow for *in vitro* experiments. *In vivo* experiment, the mice were injected with 100ng DT, then 4 ng DT per gram of body weight at 3 days interval to maintain DT ablation (21). The deletion of lung residential DCs was determined by flow cytometry.

The mice’s weight, survival rate, and lung loads were assayed following *C. psittaci* infection. In Experiment I, 96 female C57BL/6 mice were randomly divided into four groups (24 animals per group). The groups included IL-10-knockout (IL-10^−/−^), zDC-DTR, DD, and wild-type (WT) mice. Each mouse was anesthetized with 4–5% isoflurane and intranasally challenged with 1×10^5^ IFUs of EB in 20 μl of sucrose phosphate glutamate (SPG) buffer. Following treatment, the mouse body weight, activity, and survival were monitored on a daily basis. Six mice were sacrificed on days 5, 10, 15, and 20. Post-treatment, bone marrow, blood, lungs, and spleen samples were obtained for further analysis. Six lungs were removed and stored in 10% formalin to preserve the tissues. Chlamydial loads in the lungs were evaluated using a direct immunofluorescence kit (IMAGEN™; Oxoid, Cambridge, UK). IFUs were counted in 30 randomly selected Leica SP5 DM confocal microscopic fields at amplification of 400×.

IFUs/ml = the number of inclusions in 30 fields × *F* × dilution factor × 10 (to adjust for the volume of the inoculum, which was 0.1 ml). The *F* (factor) must be calibrated for each microscope (22).

$$F=\frac{Area\;of\;coverslip}{Area\;of\;30\;fields}$$

To further validate the role of IL-10 *in vivo*, IL-10 inhibitors and doxycycline (Dox; D3447; Sigma–Aldrich, Shanghai, China) were used in Experiment II. 64 mice were divided into four equal groups (16 mice per group). Mice were intranasally infected with 1×10^5^ IFUs of live EB. Subsequently, WT mice were intraperitoneally injected with ammonium trichloro(dioxoethylene-o, o’) tellurate (AS101; 10 µg per mouse, IL-10 inhibitor, 97% purity; Selleckchem, Houston, TX, USA) daily. WT mice were intraperitoneally injected with 10 µg Dox per gram of body weight. Dox is one of the widely used drugs to treat Chlamydia infection. The Dox group was used to compare the treatment effect with other control groups. The WT and IL-10^−/−^ mice were treated in a similar manner using phosphate-buffered saline (PBS). Daily monitoring of the survival outcomes of the mice was performed. Four mice from each group were sacrificed on days 5, 10, 15, and 20, and lung loads were determined.

### Determination of Cytokines and Amino Acids

The levels of interferon-gamma (IFN-γ), IL-4, IL-10, IL-12, IL-1β, and tumor necrosis factor-α (TNF-α) cytokines were measured in the sera and cellular supernatants using enzyme-linked immunosorbent assay (Invitrogen, Carlsbad, CA, USA) according to the manufacturer’s instructions.

Moreover, cells were collected and disrupted using sterile beads at 72hpi, and the supernatants and intracellular components were collected by centrifugation at 2000rpm for 20 min. The concentration of Tryptophan and Kynurenin was measured using commercial Elisa Kits (JL18438, JL18439, Jianglai Biotechnology Co., Ltd, Suzhou, China).

### Preparation of BMDC and Lung DCs

BMDCs were prepared as previously described (23). The femurs and tibias of 6–8-week-old female mice were separated, and the surrounding muscles were removed. Intact bones were placed in 75% ethanol for 2–5 min and washed with PBS. Both bone ends were removed, and the marrows were flushed with PBS. Subsequently, primary cells were cultured in RPMI-1640 (GIBCO BRL, CA, USA) cell culture medium, supplemented with streptomycin (100 μg/ml; Sigma–Aldrich, Shanghai, China), L-glutamine (2 mM, Sigma–Aldrich, Shanghai, China), 10% fetal calf serum (GIBCO BRL, CA, USA), and 10 ng/ml recombinant murine GM-CSF (315-03; PeproTech, Cranbury, NJ, USA). After cultivation for 7 days, cells were harvested and analyzed. Lymphocytic single-cell suspensions from spleens were prepared using 70-µm cell strainers (BD Falcon, NJ, USA). Cells were collected and washed twice with PBS, and red blood cells were removed using red blood cell lysis buffer (eBioscience, San Diego, CA, USA).

The lung was digested using type I collagenase (17018029; Gibco, CA, USA) and DCs were isolated by BD FACSMelody™ Cell Sorter (BD Biosciences, NJ, USA). Briefly, lung tissue was cut into 3 to 4 mm pieces using a sterile scalpel or scissors. Tissue pieces were washed 3 times with Hanks’ Balanced Salt Solution (HBSS), 50 to 200 U/ml of collagenase was added into the HBSS, incubated at 37°C for 4 h, and 3 mM CaCl_2_ was added to speed up the dissociation. Finally, cell suspensions were filtered, washed 3 times, and centrifugation was done. The final sediments were prepared using the cell culture medium for further tests. CD11c was used to detect and separate DCs according to the manufacturer’s instructions (eBioscience, San Diego, CA, USA).

### Bromodeoxyuridine (BrdU) Labeling

Splenic lymphocytes were isolated from mice by Ficoll and passed through a 70-µm cell strainer. Cells were seeded in a 96-well plate (1×10^6^ cells/well) and cultivated for 48 h. Subsequently, 1×10^6^ IFUs inactivated EBs were inoculated and incubated for 24h at 37°C in 5% CO_2_. Concanavalin A (C5275; Sigma–Aldrich, Shanghai, China) was added (5 μg/well) as a positive control, and growth medium was used as background control. All experiments were performed in triplicate. Proliferation was determined using BrdU kits (ab126556; Abcam, Cambridge, UK), and the proliferation stimulation index was calculated as the ratio between the stimulated cells and non-stimulated cells. The optical density was measured at 450nm, as previously described (24).

### Flow Cytometry Analysis

Splenic lymphocytes were centrifuged at 180×g for 10 min and the cells were collected. The cells were re-suspended in PBS to a density of 2×10^5^ cells/ml, and labelled with anti-mouse monoclonal antibodies, including CD3-PerCP-Cyanine5.5 (45-0031-82), CD4-fluorescein isothiocyanate (CD4-FITC; 11-0041-82), CD8-phycoerythrin (CD8-PE; 12-0081-81), CD11c-APC (17-0114-82), major histocompatibility complex (MHC) class II-FITC (11-5322-81), CD86-PE (12-0869-42), CD40-PE-Cyanine7 (25-0409-42), and IDO-APC (17-9477-41) (eBioscience, San Diego, CA, USA), and Annexin-V and PI (V13241; Invitrogen, Shanghai, China). For CD4 and CD8 T cell staining, cells were incubated with 1 µl of CD3-PerCP-Cyanine5.5, 2 µl of CD4-FITC, and 2 µl of CD8-PE for 25 min at room temperature in the dark. Apoptosis was evaluated by incubation with Annexin-V and PI for 30 min at room temperature. For DCs markers, cells were incubated with 1 µl of CD11c-APC, 2 µl of MHC class II-FITC, 2 µl of CD86-PE, and 2 µl of CD40-PE-Cyanine7 for 30 min at room temperature. Samples were detected using an LSR II (BD Biosciences, NJ, USA) flow cytometer and analyzed using the FlowJo software (TreeStar, NJ, USA) according to the manufacturer’s instructions.

### Statistical Analysis

All experiments were repeated at least three times. Data were expressed as mean ± standard deviation. Statistical significance was evaluated by One-Way Analysis of Variance and Tukey’s multiple comparisons of means at 95% confidence level. All data were calculated and analyzed using SPSS 22.0 software (IBM Corp., Armonk, NY, USA) and graphs were generated using the GraphPad Prism 7 software (Graphpad Software, San Diego, CA, USA). *P*-values <0.05 were considered statistically significant.

## Results

### IL-10^−/−^ Promotes Survival Following Chlamydia Infection

**Figure 1:**
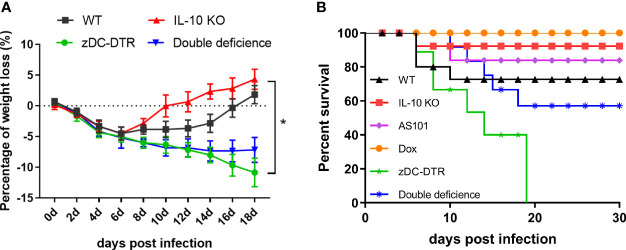
The body weight gain and survival post *Chlamydia* infection. **(A)** Body weight gains of mice in the WT, zDC-DTR, double-deficient, and IL-10^−/−^ groups were examined every 2 days post *C. psittaci* infection and the body weight of all infected mice gradually declined at 6 dpi; the body weight of zDC-DTR and DD mice continued to decline up to 14 dpi; DD mice gradually attained normal body weight after day 14, whereas zDC-DTR mice did not. **(B)** Monitoring of the survival of mice up to 30 dpi. More than 90% of IL-10^−/−^ mice, 85% of AS101-treated mice, 70% of WT mice, and 60% of DD mice were alive after 30 days. All zDC-DTR mice died, whereas all mice in the Dox group survived during the observation period. Differences were analyzed by ANOVA (*P<0.05).

DD mice were generated by breeding IL-10^−/−^ mice with zDC-DTR mice, and the genotypes were confirmed by polymerase chain reaction (**Supplemental Figure S1**). DCs deletion was validated in the mouse model with DT treatment before the experiments (**Supplemental Figure S2A**). All mice showed obvious clinical symptoms following infection with *C. psittaci*, including reduced activity and loss of body weight. Body weight in all infected mice gradually declined at 6 days post-inoculation (dpi). zDC-DTR and DD mice showed a continuous reduction in body weight at 14 dpi, and DD mice recovered slowly after day 14. IL-10^−/−^ mice and WT mice recovered body weight at 12 dpi and 16 dpi, respectively. However, zDC-DTR mice did not show recovery of body weight (**Figure 1A**). More than 90% of IL-10^−/−^ mice, 85% of AS101-treated mice, 70% of WT mice, and 60% of DD mice were alive at 30 dpi. Notably, all zDC-DTR mice died, while all mice in the Dox group survived (**Figure 1B**).

### IL-10^−/−^ Mice Had Fewer Lung Lesions and Reduced Bacterial Loads

**Figure 2:**
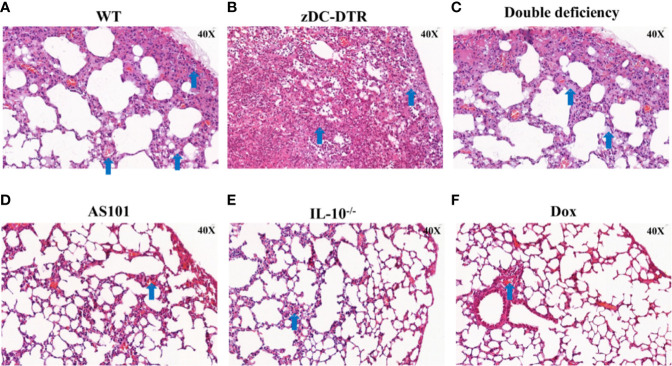
Lung lesions post Chlamydia infection. Compared with lesions in WT mice **(A)**, severe lung lesions were observed in zDC-DTR mice **(B)**, characterized as more pulmonary hemorrhage and albuminoidal secretion while alveolar interstitial inflammations were moderate in DD mice **(C)**. The lungs of DD mice displayed severe alveolar interstitial damage along with slight hemorrhage and protein-like infiltrations. Less lung lesions were observed among the AS101 **(D)**, IL-10^−/−^ groups **(E)**, and Dox **(F)** without significant differences. The image magnification is 400×. Blue arrows point to the characteristic lesions in each panel.

**Figure 3:**
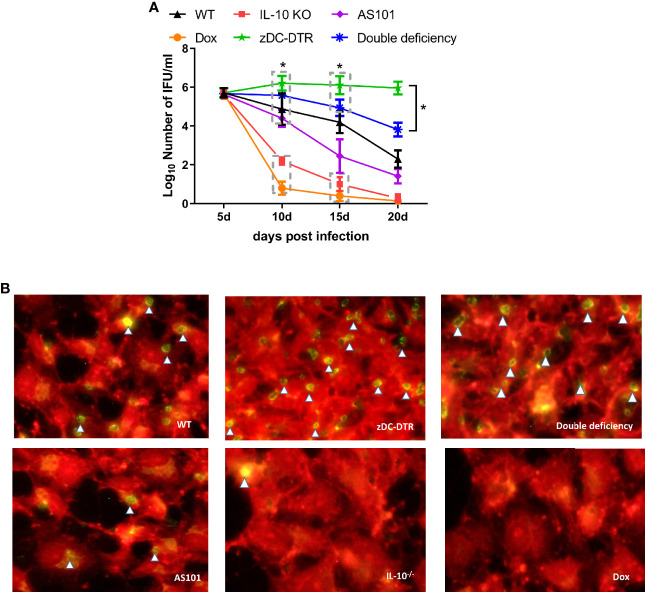
Lung loads post *Chlamydia* infection. **(A)** Calculations of *C. psittaci* loads at 5, 10, 15, and 20 dpi using direct immunofluorescence. Data were expressed as the mean ± SD (n=8 mice per group). Lower bacterial loads were found in the Dox and IL-10^−/−^ groups compared with those of the zDC-DTR, DD, WT, and AS101 groups from day 10 to day 15. Afterward, high bacterial clearances were observed in the AS101 and IL-10^−/−^ groups. The IFUs of DD mice were significantly reduced compared with those of zDC-DTR mice at 20 dpi. **(B)** Direct immunofluorescence staining of *Chlamydia* inclusion bodies at 20 dpi. The green dots (white arrow) showed *Chlamydia* inclusion bodies. Differences were analyzed by ANOVA (*P<0.05).

Post mortem analysis revealed fewer lesions in the lungs of IL-10^−/−^ mice compared with the thickened alveolar walls in WT mice (**Figure 2A**). Compared with WT mice, zDC-DTR mice displayed severe lung lesions, characterized by extensive pulmonary hemorrhage and albuminoidal secretion associated with inflammatory cell infiltration and necrosis (**Figure 2B**). Thickened alveolar septum, slight hemorrhages, and protein-like infiltration were observed in the lungs of DD mice (**Figure 2C**). There were no significant differences observed among AS101, Dox, and IL-10^−/−^ groups (**Figures 2D–F**). IFUs were calculated by direct immunofluorescence. High chlamydial loads were observed at 5 dpi, while lower bacterial loads were observed in the Dox and IL-10^−/−^ groups compared with the zDC-DTR, DD, WT, and AS101 groups from day 10 to 15. High bacterial clearances were observed in the AS101 and IL-10^−/−^ groups. IFUs were significantly reduced in DD mice compared with zDC-DTR mice at 20 dpi (**Figure 3A**). Fewer IFUs were detected in the AS101 and IL-10^−/−^ groups compared with the zDC-DTR and DD groups at 20 dpi (**Figure 3B**).

### IL-10^−/−^ Enhances the Proliferation and Differentiation of T Cell Subsets and Cytokine Secretion

**Figure 4:**
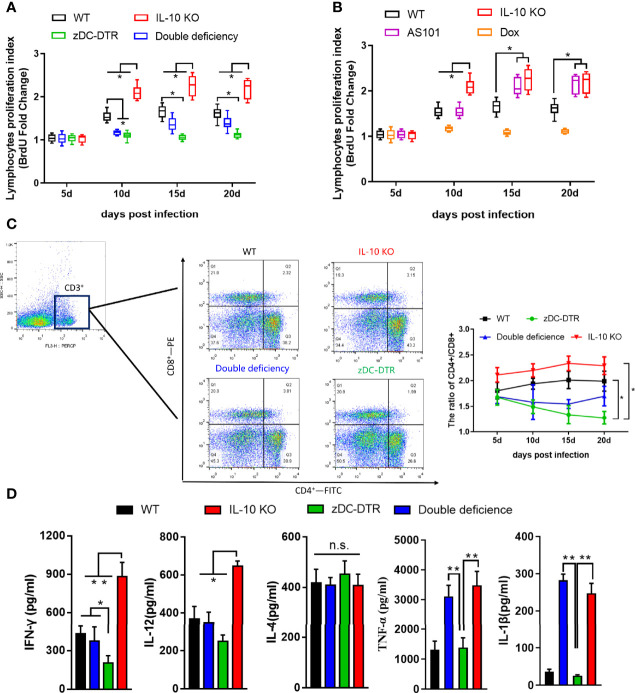
Lymphocyte proliferation, T cell subsets, and cytokines in sera post infection. **(A)** BrdU assay detection of lymphocyte proliferation in Experiment I. Compared with WT mice, lymphocyte proliferation was significantly decreased in DD mice at 10 dpi. Afterward, lymphocyte proliferation gradually increased in DD mice to the levels observed in WT mice. **(B)** Higher lymphocyte proliferations were determined in AS101-treated mice compared with those of WT mice from day 15 to day 20. **(C)** Flow cytometry examination of T cell subsets (CD3-PE-cy5, CD4-FITC, and CD8-PE) at 5, 10, 15, and 20 dpi. CD4 and CD8 were analyzed by pre-gated CD3^+^ cells. A higher proportion of CD4^+^/CD8^+^ was observed in IL-10^−/−^ and WT mice compared with zDC-DTR mice from 15 to 20 dpi. However, the T cell subset ratio in DD mice began to gradually increase at 15 dpi and showed no significant difference from that of WT mice at 20 dpi. **(D)** Cytokines (IFN-γ, IL-12, IL-4, TNF-α, and IL-1β) in sera were detected using commercial ELISA kits. All data were expressed as the mean ± SD (n=5 sera per group). Differences were analyzed by ANOVA (*P<0.05, **P<0.01, n.s., no statistical significance).

There were no differences in lymphocyte proliferation among the four groups at 5 dpi. However, compared with the other groups, higher lymphocyte proliferation was observed in the IL-10^−/−^ group at 10–20 dpi. Compared with WT mice, there was a significant decrease in lymphocyte proliferation in DD mice at 10 dpi. Later, the lymphocyte proliferation in DD mice was comparable to WT mice (**Figure 4A**). There was a significant increase in lymphocyte proliferation in AS101-treated mice, compared with WT mice at 15–20 dpi (**Figure 4B**). A higher proportion of CD4^+^/CD8^+^ T cells was observed in IL-10^−/−^ and WT mice than zDC-DTR mice from 15 to 20 dpi. However, the T cell subset ratio in DD mice gradually increased from 15 dpi and 20 dpi, but these findings did not differ from those of WT mice (**Figure 4C**). To validate the proportions of CD4^+^ and CD8^+^ T cells in the lungs, higher proportions were observed in the IL-10^-/-^ group and DD group compared to the zDC-DTR group and WT group (**Supplemental Figure S2B**).

At 20 dpi, there was a significant increase in the levels of IFN-γ and IL-12 cytokines in IL-10^−/−^ mice and DD mice compared with zDC-DTR mice. However, there were no significant differences between WT and DD mice. The levels of TNF-α and IL-1β dramatically increased in the IL-10^−/−^ and DD groups compared with the zDC-DTR and WT groups. Moreover, the levels of IL-4 did not differ among any of the groups (**Figure 4D**).

### IL-10^−/−^ Facilitates DC Maturation and T Cell Activation *In Vitro*

**Figure 5:**
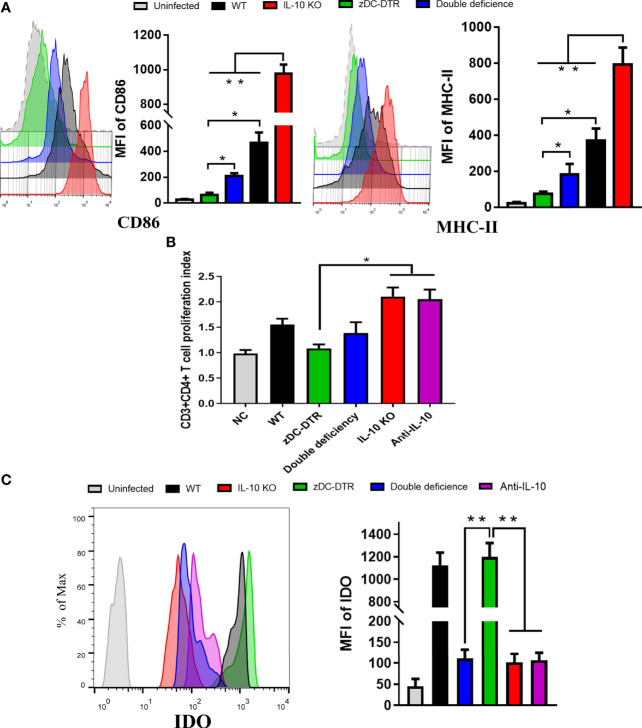
DCs maturation, T cell proliferation, and IDO expression in the co-culture system. **(A)** Flow cytometry detection of CD86 and MHC-II DC surface markers; mean fluorescence intensity (MFI) was calculated at 72 hpi. CD86 and MHC-II were analyzed by pre-gated CD11c^+^ cells, CD11c was gated by isotype. CD86 and MHC-II surface markers were significantly upregulated in the co-culture system of CD4^+^T cells and IL-10^−/−^DCs. Compared to the DCs from the zDC-DTR group, CD86 and MHC-II were significantly higher in the DD group. **(B)** Measurement of CD4^+^ T cell proliferation *via* BrdU assay in the co-culture system of CD4^+^T cells and DCs at 72 h. Higher CD4^+^ T cell proliferation was observed in the IL-10^−/−^ and anti-IL-10 groups than the zDC-DTR group (*P*<0.05). There were no statistically significant differences observed between the DD, IL-10^−/−^, and anti-IL-10 groups. **(C)** Flow cytometry detection of IDO expression; MFI was calculated at 72 h. Significantly increased IDO expression was observed in BMDCs from the zDC-DTR and WT mice. Differences were analyzed by ANOVA (*P<0.05, **P<0.01).

CD86 and MHC-II surface markers were significantly upregulated in the IL-10^−/−^ DC group compared with the other groups. However, CD86 and MHC-II were highly upregulated in the DD group compared with the zDC-DTR group (**Figure 5A**). After co-cultivation of DCs with T cells, increased CD4^+^ T cell proliferation was observed in the IL-10^−/−^ and anti-IL-10 groups compared with the zDC-DTR group. However, there was a significant difference in cell proliferation between the DD and WT groups (**Figure 5B**).

Flow cytometry analysis was used to determine IDO expression in the co-culture system of CD4^+^ T cells and DCs. DCs were isolated from IL-10^−/−^, zDC-DTR, DD, and WT mice, respectively. A significant increase in IDO expression was observed in zDC-DTR and WT groups at 72 h post infection (hpi) (*P*<0.01). However, there was no significant difference between the zDC-DTR and WT groups. IDO expression was downregulated in anti-IL-10 and DD groups, indicating that IDO was significantly downregulated in the absence of IL-10 DCs (**Figure 5C**). To validate IDO expression in the lungs, the DCs were isolated from lung tissues, and their proportion was found to account for 3% of the lung cells. IDO expression was found to significantly increase in both zDC-DTR and WT mice. However, decreased expressions of IDO were found in DD mice, IL-10^−/−^ mice, and anti-IL-10 mice (**Supplemental Figure S4**). As a key enzyme, IDO catalyzes the conversion of Tryptophan to Kynurenine. DD, IL-10 KO, and Anti-IL-10 groups showed a significant decrease in the production of Kynurenin. The ratio of Tryptophan/Kynurenin was dramatically increased compared with WT and zDC-DTR groups (**Supplemental Table S1**). Taken together, these results indicated that the expression and activity of IDO were inhibited in absence of IL-10.

### IL-10^−/−^ Activated the Expression of the OX40L Receptor and Th1 Cytokines in the Co-Culture System

**Figure 6:**
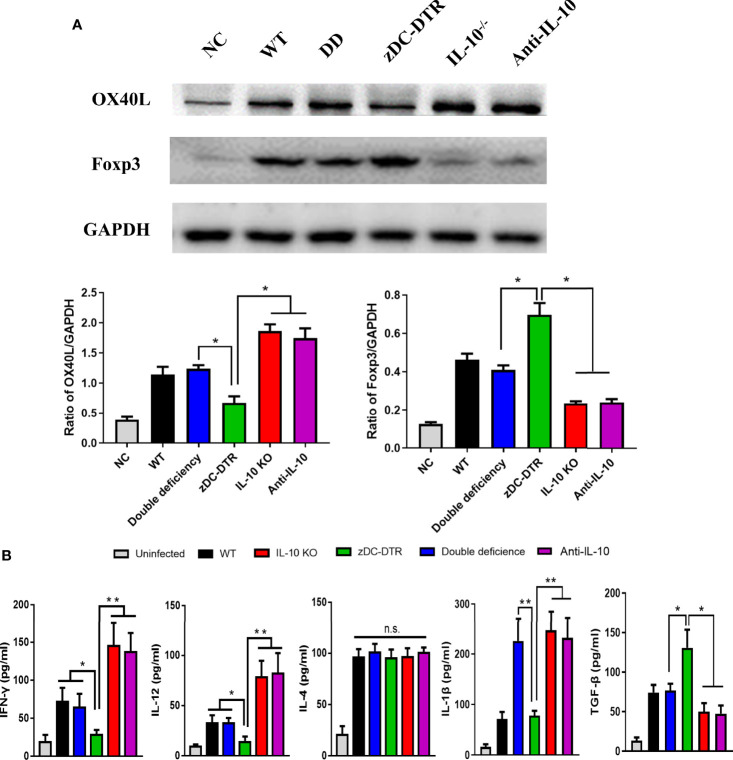
Detection of the OX40L receptor, Treg expression, and cytokine secretion in the co-culture system. **(A)** Lysis of BMDCs using NP40 reagents, and detection of OX40 ligand (OX40L) and FOXP3 expression by Western blotting. GAPDH was used as the internal control. Higher OX40L expression was observed in the IL-10^−/−^, anti-IL-10, and DD groups than the zDC-DTR group (*P*<0.05). In contrast to OX40L, higher levels of FOXP3 were observed in the zDC-DTR group than the other groups at 72 hpi (*P*<0.05). **(B)** Quantification of the levels of cytokines (IFN-γ, IL-12, IL-4, TGF-β, and IL-1β) in the supernatants using commercial ELISA kits at 72 hpi. All data were expressed as the mean ± SD (n=5 per group). Higher IFN-γ and IL-12 levels were observed in the IL-10^−/−^, anti-IL-10 (*P*<0.01), and DD groups (*P*<0.05) than the zDC-DTR group. Moreover, higher TGF-β levels were detected in the zDC-DTR group compared to other groups. Significantly higher IL-1β levels were observed in the IL-10^−/−^, the anti-IL-10, and the DD groups compared to the WT and the zDC-DTR groups. No significant differences were determined in IL-4 secretions among the groups. The differences were analyzed by ANOVA (*P<0.05, **P<0.01, n.s., no statistical significance).

Higher OX40L levels were detected in the IL-10^−/−^, anti-IL-10, and DD groups compared with the zDC-DTR group (*P*<0.05). Significantly higher levels of forkhead box P3 (FOXP3) were observed in the zDC-DTR group compared with other groups at 72 hpi (**Figure 6A**). Moreover, a similar trend of Foxp3 expression was revealed using data from flow cytometry (**Supplemental Figure S5**). Furthermore, OX40L siRNA was used to block the interaction of OX40-OX40L pathway. The Foxp3 expression was significantly increased in Treg cells, and the OX40L-blocked group showed increased productions of the immunosuppressive factor TGF-β and IL-10 (**Supplemental Figures S6A, B**). Besides, the Chlamydia burdens were significantly increased after OX40L siRNA treatment compared with the mock siRNA group (**Supplemental Figure S6C**).

*In vitro* analysis of co-culture, higher levels of IFN-γ and IL-12 in the IL-10^−/−^, anti-IL-10 (*P*<0.01), and DD groups (*P*<0.05) were determined compared with the zDC-DTR group at 72 hpi. Additionally, higher levels of TGF-β expression were detected in the zDC-DTR group compared with other groups. A significant increase in IL-1β levels was observed in the IL-10^−/−^, anti-IL-10, and DD groups compared with WT and zDC-DTR groups. Finally, there were no significant differences in IL-4 levels among all the groups at 72 hpi (**Figure 6B**).

### IL-10^−/−^ Promoted the Assembly of the NLRP3 Inflammasomes Leading to DCs Apoptosis

**Figure 7:**
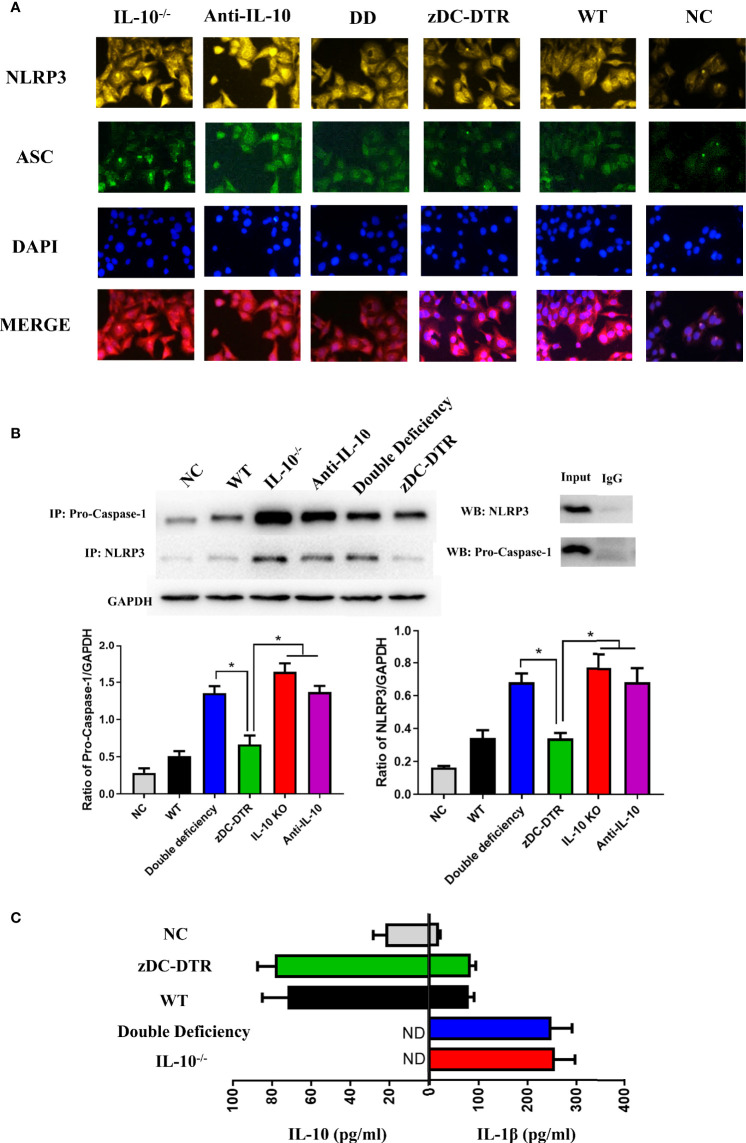
Activation and assembly of the NLRP3 inflammasome. **(A)** BMDCs were fixed with methanol and incubated with anti-NLRP3 (yellow), anti-ASC (green), and DAPI (blue) at a multiplicity of infection (MOI) of *C. psittaci* at 72 hpi. The relative fluorescence intensity was detected co-localization. Co-localization of NLRP3 and ASC was observed more frequently in the IL-10^−/−^, anti-IL-10, and DD groups than the zDC-DTR group (*P*<0.05). **(B)** The immunoprecipitation of NLRP3 and pro-caspase 1 was assessed by Western blotting; the ratio of pro-caspase 1 and NLRP3 was calculated to determine the expression levels. Co-immunoprecipitation of NLRP3 and pro-caspase 1 was higher in the IL-10^−/−^, the anti-IL-10, and the DD groups than the zDC-DTR group. **(C)** Secretions of IL-10 and IL-1β were quantified in the supernatants of the co-culture system of CD4^+^T cells and DCs at 72 hpi using commercial ELISA kits. All data were expressed as the mean ± SD (n=5 per group). The differences were analyzed by ANOVA (*P<0.05, ND, no detection). IL-10 secretion was negatively correlated with the levels of IL-1β in all groups.

At 72 hpi, co-localization of NLRP3 and apoptosis-associated speck-like protein containing a CARD (ASC) was higher in the IL-10^−/−^, anti-IL-10, and DD groups compared with the zDC-DTR group (**Figure 7A**). Moreover, NLRP3 and pro-caspase 1 co-immunoprecipitation was higher in IL-10^−/−^, anti-IL-10, and DD groups, and these findings were comparable to NLRP3 and ASC co-localization following treatment (**Figure 7B**). IL-10 secretion was negatively correlated with the levels of IL-1β in all groups (**Figure 7C**).

**Figure 8:**
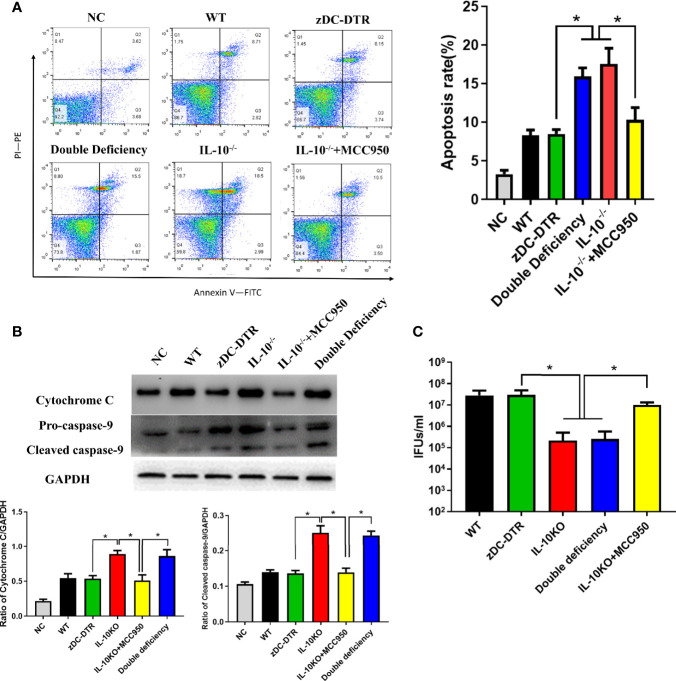
Activation of DC apoptosis was upregulated in IL-10^−/−^ DCs via NLRP3 inflammasome pathway. **(A)** Flow cytometry detection of Annexin-V-FITC- and PI-PE-positive BMDCs with an MOI of *C. psittaci* at 72 hpi. The four quadrants were gated by negative control. Significant higher apoptosis was observed in the IL-10^−/−^ and the DD groups compared to the WT and the zDC-DTR groups. Treatment with the NLRP3-specific inhibitor MCC950 lowered apoptosis compared with that of the DD and IL-10^−/−^ groups. **(B)** The levels of cytochrome C and pro-caspase 9 were determined by Western blotting. Both cytochrome C and pro-caspase 9 were significantly upregulated in the IL-10^−/−^ and the DD groups compared with the zDC-DTR group. **(C)** Lysis of cells *via* ultra-sonication. Infectious progeny EBs were counted using IFU assay. All data were expressed as the mean ± SD (n=5 per group). The differences were analyzed by ANOVA (*P<0.05).

Significant increase of apoptosis was observed in the IL-10^−/−^ and DD groups compared with the WT and zDC-DTR groups. In the present study, the specific NLRP3 inhibitor, MCC950 significantly reduced apoptosis (**Figure 8A**). The levels of cytochrome C and pro-caspase 9 were significantly higher in the IL-10^−/−^ and DD groups compared with the zDC-DTR group (**Figure 8B**). Additionally, higher chlamydial clearance was observed in the IL-10^−/−^ and DD groups compared with the MCC950, zDC-DTR, and WT groups. There was no significant difference in chlamydial clearance among the MCC950, zDC-DTR, and WT groups (**Figure 8C**).

## Discussion

*C. psittaci* is a typical intracellular pathogen with multiple transmission pathways in both animals and humans, and causes severe respiratory disease (1). Currently, there is a lack of efficient vaccines against *C. psittaci* infections. Therefore, it is important to understand the interaction between host cells and the pathogen, in particular, the IL-10-mediated function of DCs during *C. psittaci* infection.

In the present study, mortality, lymphocyte proliferation, cytokine secretion, pathology, and bacterial loads were examined in IL-10^−/−^, zDC-DTR, and DD mice. Varying survival levels were observed in different groups. The observed mortality rates were 40%, 100%, and 10% for DD, zDC-DTR, and IL-10^−/−^ groups, respectively. Lung examination revealed fewer lesions and higher bacterial clearance in DD mice compared with zDC-DTR mice, suggesting that IL-10 and DCs played an important role in *C. psittaci* infection and IL-10 might regulate DCs function and affect the mice survival rate. Interestingly, the lung lesions after treatment with AS101 were comparable to those in IL-10^−/−^ mice, indicating that the IL-10 inhibitor may be a therapeutic candidate for combating *C. psittaci* infection.

Our results showed that co-cultivation of BMDCs from IL-10^−/−^, zDC-DTR, and DD mice with CD4^+^ T cells led to DCs maturation, T cell proliferation, and upregulation of the OX40-OX40L receptor and NLRP3 inflammasome. We also observed that IDO expression was downregulated and Tregs were reduced in the DD group compared with the zDC-DTR group. Additionally, the levels of cytochrome C and pro-caspase 9 were higher in DD-DCs and IL-10^−/−^ DCs than in zDC-DTR group, leading to increased apoptosis and chlamydial clearance. Maturation and activation of IL-10^−/−^ DCs were regulated by OX40-OX40L, activation of T cells and the NLPR3 inflammasome, downregulation of IDO expression, and reduction in Tregs, leading to enhanced apoptosis through the release of cytochrome C and pro-caspase 9.

The mechanism by which IL-10 influences DCs function is unclear. IL-10-mediated DCs function is inhibited by binding of March-I to MHC-II *via* CD83 and downregulation of MHC-II in activating DCs in a March-I-dependent manner. Moreover, Tregs secrete IL-10 that induces the expression of March-I by downregulating MHC-II and CD86 on the surface of DCs (25). In the present study, zDC-DTR mice displayed high mortality and severe lung lesions, whereas DD mice showed a 60% survival rate and mild pathology. IL-10^-/-^ promotes the function of deficient DC, thus, the DD group was comparable to the WT group. Our findings were consistent with those of previous studies on *Chlamydia trachomatis*, *Chlamydia muridarum* (26), and *Chlamydia pneumoniae* infections (27). Similarly, IL-10 deficiency in mice facilitated DCs antigen-presentation and Th1 immune response (28). Furthermore, activated DCs induces the activation of T cells. In our study, IL-10 knockout significantly promoted the proliferation of T lymphocytes *in vivo* and *in vitro*. We also measured the ratio of CD4^+^ and CD8^+^ T cells, representing the host immune homeostasis. The ratio of zDC-DTR showed a continuous decline from 5 dpi to 20 dpi, which was lower than in other groups, indicating that the immune balance was altered to immunosuppression. IL-10 knockout group showed highly induced differentiation of CD4^+^ T cells and enhanced immune response.

We examined the receptors involved in DCs activation, and found that the OX40 tumor necrosis factor receptor and its ligand OX40L were upregulated in DD-DCs, IL-10^−/−^ DCs, and anti-IL-10-treated DCs in the co-culture system of CD4^+^T cells and DCs. Together, these results imply that the function of IL-10^−/−^ DCs are activated by OX40-OX40L receptors. Previous studies demonstrated that OX40 was expressed on the surface of Tregs, while OX40L was presented on DCs. The interaction between OX40 and OX40L plays a dominant anti-tumor role. OX40- or OX40L-deficient mice have distinctly reduced CD4^+^ T cell propagation (29). Moreover, transfection of OX40L mRNA into DCs promotes CD4^+^ T cell activation and polarization (30). Additionally, OX40-OX40L interaction induces the secretion of effector cytokines, prolongs T cell activation, and contributes to Th1 response by regulating Tregs (31). It is the first time that OX40-OX40L activation has contributed to CD4^+^ T cell proliferation and Th1 immune response through inhibition of Tregs. Therefore, OX40L may be a potential therapeutic target for Chlamydia or other microbial infections.

Co-localization of NLRP3 and ASC, and co-immunoprecipitation of NLRP3 and pro-caspase 1 were upregulated following T cell co-cultivation with DCs from the DD and the IL-10^−/−^ mice. Consequently, higher apoptosis levels were observed in the two groups. Our results indicate that IL-10^−/−^ DCs promote the activation and assembly of NLRP3 inflammasome and facilitates apoptosis, resulting in chlamydial clearance. This is consistent with a previous report showing that the *Chlamydia abortus* PmpD18.1 activates the NLRP3 inflammasome and induces the secretion of IL-1β and IFN-γ (18). To validate the results, and NLRP3 specific inhibitor, MCC950, and anti-IL-10 antibody were used to determine the rate of apoptosis through the IL-10^−/−^/NLRP3 inflammasome/DCs apoptosis signaling pathway. Interestingly, the secretion of IL-1β was negatively correlated with IL-10 expression, indicating that the NLRP3 inflammasome was regulated by IL-10 and not DCs depletion. Our findings are inconsistent with those from previous reports showing that NLRP3 inflammasome assembly is suppressed in IL-10^−/−^ DCs *via* inhibition of the P2X purinoceptor 7 receptor (P2X7R) and reduction in the levels of intracellular Ca^2+^, leading to inhibition of DCs apoptosis post infection with *C. muridarum* (32). The differences in NLRP3 inflammasome following infection with different Chlamydia species have been documented, suggesting that IL-10^−/−^ has a specific effect on NLRP3-mediated apoptosis.

High IDO expression was observed in DCs of WT and zDC-DTR mice, suggesting that it is inversely regulated by IL-10. However, the mechanism involved in the interaction between IDO and IL-10 remains elusive. As a critical immunosuppressive factor, IDO is generally expressed in tumor cells and immunosuppressive cells. Increasing levels of IDO allow cancer cells to evade immune killing since it is activated in the mouse lung and facilitates bacterial invasion following *Chlamydia muridarum* and *Chlamydia pneumonia* infection (33). Moreover, the expression of TGF-β and Foxp3 correlates with IDO production during infection with *Chlamydia trachomatis* (34). Blocking IDO activity increases the susceptibility of cells to Dox treatment (35). IFN-γ has antiviral activity against poxviruses, as well as numerous other viruses, bacteria, and parasites. IDO mediates the antiviral activity of IFN-γ against virus, while the L-tryptophan IDO inhibitor completely blocks the antiviral activity of IFN-γ (36). In this study, higher levels of IFN-γ and IL-12 were observed in DD and IL-10^−/−^ mice compared with zDC-DTR mice. These results were corresponded with the role of T cell proliferation in the Th1 immune response. A recent study reported high levels of IL-10, decreased IL-12p70 and IFN‐γ levels, and an elevated Treg population in a *Helicobacter pylori*-pulsed co-culture system of CD4^+^T cells and DCs *in vitro* (37). Other cells also produce IL-10, for example, innate immune cells such as macrophages and NK cells express IL-10 in response to microbial stimuli through TLR or NOD ligands. Adaptive immune cells such as Th2, Th17, and Treg express IL-10 were stimulated by a variety of cytokines. Therefore, the regulatory network in the host is complicated, and this study provides partial mechanisms of the interaction between IL-10 and DCs. However, to explain the precise underlying molecular processes, these mechanisms should be further elucidated.

**Figure 9:**
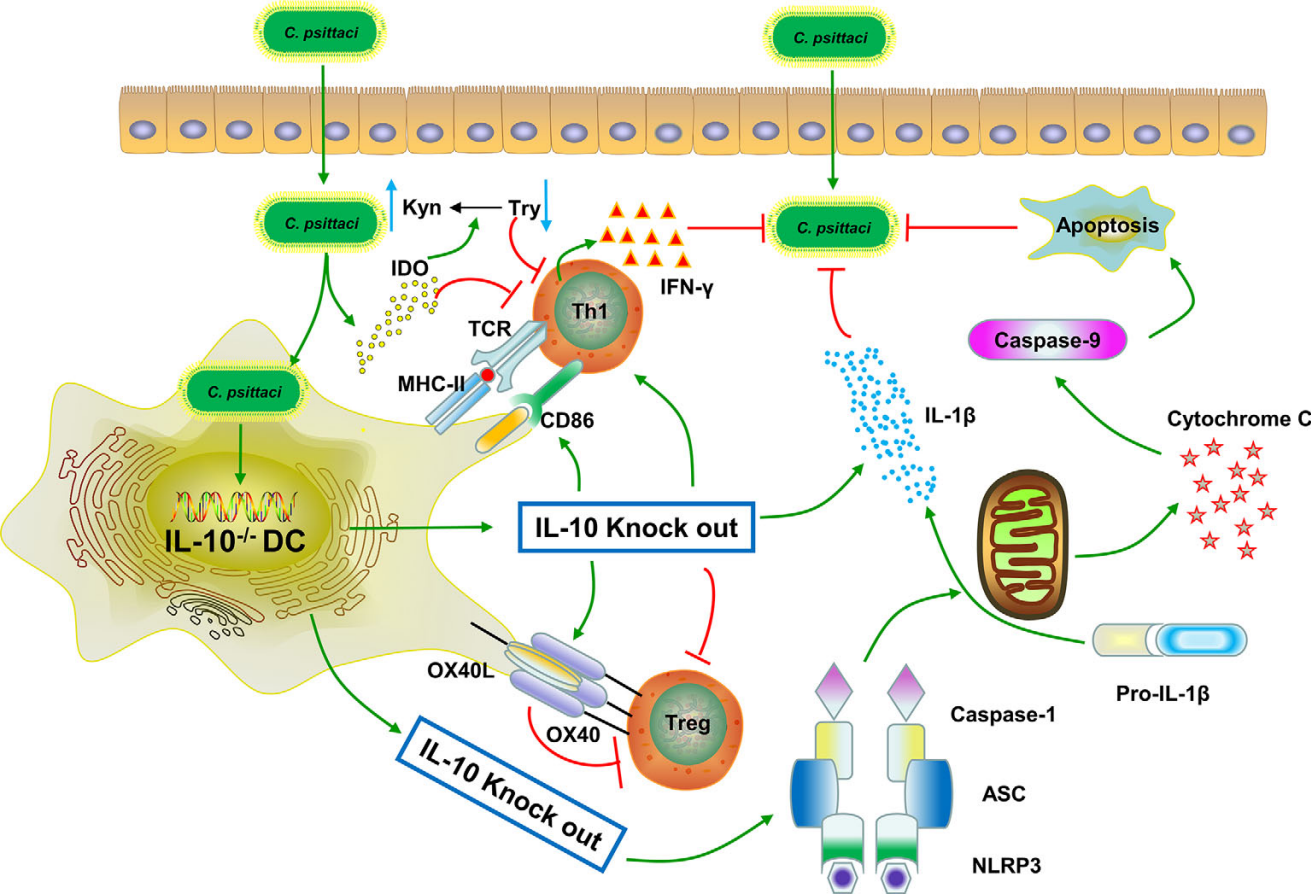
Possible interaction between IL-10 and DCs during *C. psittaci* infection. IL-10 knockout enhanced DC maturation, T cell proliferation, and increased Th1 cytokine secretion during *C. psittaci* infection. IL-10^−/−^ improved the function of the deficient DCs by upregulating the OX40-OX40L, increasing CD4^+^ T cell proliferation and NLPR3 inflammasome assembly, downregulating IDO expression, and reducing Tregs, leading to DCs apoptosis and chlamydial clearance. The green line arrows denote promotion while the red line arrows denote suppression.

In conclusion, this study utilized gene-deficient mice and a BMDC model to investigate the relationship between IL-10 and DCs during *C. psittaci* infection. DD mice were firstly generated to investigate the interaction between IL-10 and DCs. Besides, enhanced DCs maturation and elevation of Th1 cytokines were observed in IL-10^−/−^ DCs. These results showed that IL-10^−/−^ might improve the function of deficient DCs through the upregulation of OX40-OX40L, CD4^+^ T cell proliferation and assembly of the NLPR3 inflammasome, downregulation of IDO expression, and reduction of Tregs, leading to high survival rates and chlamydial clearance (**Figure 9**). This study highlights the interaction between IL-10 and DCs, and reveals that IL-10, OX40-OX40L, and the NLPR3 inflammasome are potential therapeutic targets for the treatment of *C. psittaci* infection. The study also expands our understanding of the pathogenesis of *C. psittaci* infection.

## Data Availability Statement

The original contributions presented in the study are included in the article/**Supplementary Material**. Further inquiries can be directed to the corresponding author.

## Ethics Statement

The animal study was reviewed and approved by the Committee on Experimental Animal Management of China Agricultural University.

## Author Contributions

CH provided the study concept and design. QL performed the experiments, analyzed the data, and wrote the manuscript. HQ, GQ, and ZS assisted with animal experiments. XL and YW assisted experiments. All authors contributed to the article and approved the submitted version.

## Funding

This study was funded by the National Natural Science Foundation of China (Grant No. 31672517) and Taishan Scholar Project of Shandong province (Grant No. ts201511084).

## Conflict of Interest

The authors declare that the research was conducted in the absence of any commercial or financial relationships that could be construed as a potential conflict of interest.

## Supplementary Material

Supplementary Table 1 The ratio of Tryptophan and Kynurenine cells were collected and disrupted by sterile beads at 72h, the supernatants and intracellular components were centrifugated at 2000 rpm for 20 min. The concentrations of Tryptophan and Kynurenin were measured using commercial Elisa Kits, ratio and P value were calculated. The activity of IDO was comparable to IDO expression. Concentrations of Kynurenin were reduced significantly in the DD, IL-10 KO, and anti-IL-10 groups, indicating the decreased activities of IDO in absence of IL-10. Significant differences were analyzed by *t*-test (**P*<0.05, ***P*<0.01).

Supplementary Figure 1 PCR identification of mouse genotypes. The specific genes were verified by PCR according to the protocols of Jackson Laboratory. **(A)** IL-10 mutant (002250) target band was roughly 312bp. **(B)** DC mutant (019506) target band was roughly 420bp. NC, negative control.

Supplementary Figure 2 DCs deletion efficiency, CD4^+^ and CD8^+^ T cells infiltration in the lungs. **(A)** Mice were euthanized after DT treatment; lungs were collected and digested to a single cell. DCs were labeled with the markers of CD11c-APC, CD86-PE, and MHC-II-FITC to verify deletion of lung residential DCs. CD86 and MHC-II were analyzed by pre-gated CD11c^+^ cells, CD11c was gated by isotype. **(B)** Mice were euthanized at 7 dpi and lungs were collected and digested to prepare a single cell. CD4-FITC and CD8-PE were labeled to determine the infiltration in the lungs. CD4 and CD8 were analyzed by pre-gated CD3^+^ cells.

Supplementary Figure 3 The isotype control of flow cytometry. **(A)** Detecting isotype of CD4+ and CD8+ T lymphocyte (CD4-FITC, CD8-PE). **(B)** Detecting IDO isotype (IDO-APC). **(C)** The negative control of apoptosis (Annexin V-FITC, PI-PE). Flow cytometry refers to isotype to set gates for further analysis.

Supplementary Figure 4 IDO expression in the lungs by Western blotting. **(A)** Lung residential DCs were isolated by BD FACSMelody™ Cell Sorter. The proportion of DCs was roughly 3% of lung cells. **(B)** Western blotting detection of IDO expression in the lung at 72 hpi. IDO expression increased significantly in DCs from the zDC-DTR mice and WT mice (**P*<0.01). On contrary, IDO expression was decreased obviously in the DD mice, the IL-10^−/−^ mice, and the anti-IL-10 mice.

Supplementary Figure 5 Detection of Foxp3 expression by flow cytometry. **(A)** Cells were collected and stained with surface markers (CD4), then fixed and permeabilized. This was followed by incubation with a nuclear antibody (Foxp3). Finally, cells were stained with fluorescent secondary antibodies. Goat anti-rabbit IgG-PE was used as the secondary antibody at 1/2000 dilution. Foxp3 expression was significantly decreased in the IL-10-/- group and Anti-IL-10 group compared to the zDC-DTR group. A similar trend was comparable to the expression by Western blotting.

Supplementary Figure 6 siRNA blocked the OX40-OX40L pathway. **(A)** Co-culture system was treated OX40L siRNA and mock siRNA in 24-well plate, respectively. After 24h treatment, cells were infected at an MOI of *C. psittaci*. At 72 hpi, cells were collected and assayed for the expression of OX40L and Foxp3 by Western blotting. The expression of Foxp3 significantly increased after OX40L siRNA treatment. **(B)** TGF-β and IL-10 were collected and determined by ELISA kits in the co-culture system supernatants. Higher expression of the TGF-β and IL-10 were found in the OX40L blocked group. **(C)** After 24h siRNA treatment, cells were infected at an MOI of *C. psittaci*. Post 72h infection, cells were collected and lysed by ultra-sonication, infectious progeny EBs were counted using IFU assay. All data were expressed as the mean ± SD (n=5 per group). The differences were analyzed by ANOVA (**P*<0.05).
