# Supplementary material for: IL-10−/− Enhances DCs Immunity Against Chlamydia psittaci Infection via OX40L/NLRP3 and IDO/Treg Pathways
Source: Front Immunol. 2021 May 21;12:645653. doi: 10.3389/fimmu.2021.645653 (PMC8176032; doi:10.3389/fimmu.2021.645653)
Supplement: Supplementary Table 1 — The ratio of Tryptophan and Kynurenine cells were collected and disrupted by sterile beads at 72h, the supernatants and intracellular components were centrifugated at 2000 rpm for 20 min. The concentrations of Tryptophan and Kynurenin were measured using commercial Elisa Kits, ratio and P value were calculated. The activity of IDO was comparable to IDO expression. Concentrations of Kynurenin were reduced significantly in the DD, IL-10 KO, and anti-IL-10 groups, indicating the decreased activities of IDO in absence of IL-10. Significant differences were analyzed by t-test (*P<0.05, **P<0.01). [file Table_1.docx]

**Supplemental Table S1**

| Group | *n* | C_Trp_(μmol/L) | C_Kyn_(μmol/L) | C_Trp_/C_Kyn_ | *P* |
| --- | --- | --- | --- | --- | --- |
| Wild Type | 5 | 65.06±2.92 | 11.90±0.89 | 5.48±0.33 | — |
| zDC-DTR | 5 | 64.36±2.08 | 13.36±1.78 | 4.86±0.61 | 0.220 |
| Double Deficience | 5 | 65.01±1.72 | 1.69±0.18 | 38.61±3.81 | 0.004** |
| IL-10 KO | 5 | 62.12±2.30 | 1.70±0.27 | 37.11±6.33 | 0.012* |
| Anti-IL-10 | 5 | 62.99±1.41 | 1.47±0.17 | 42.88±3.90 | 0.003** |

**The ratio of Tryptophan and Kynurenin**
