# Supplementary figures and images for: IL-10−/− Enhances DCs Immunity Against Chlamydia psittaci Infection via OX40L/NLRP3 and IDO/Treg Pathways
Source: Front Immunol. 2021 May 21;12:645653. doi: 10.3389/fimmu.2021.645653 (PMC8176032; doi:10.3389/fimmu.2021.645653)

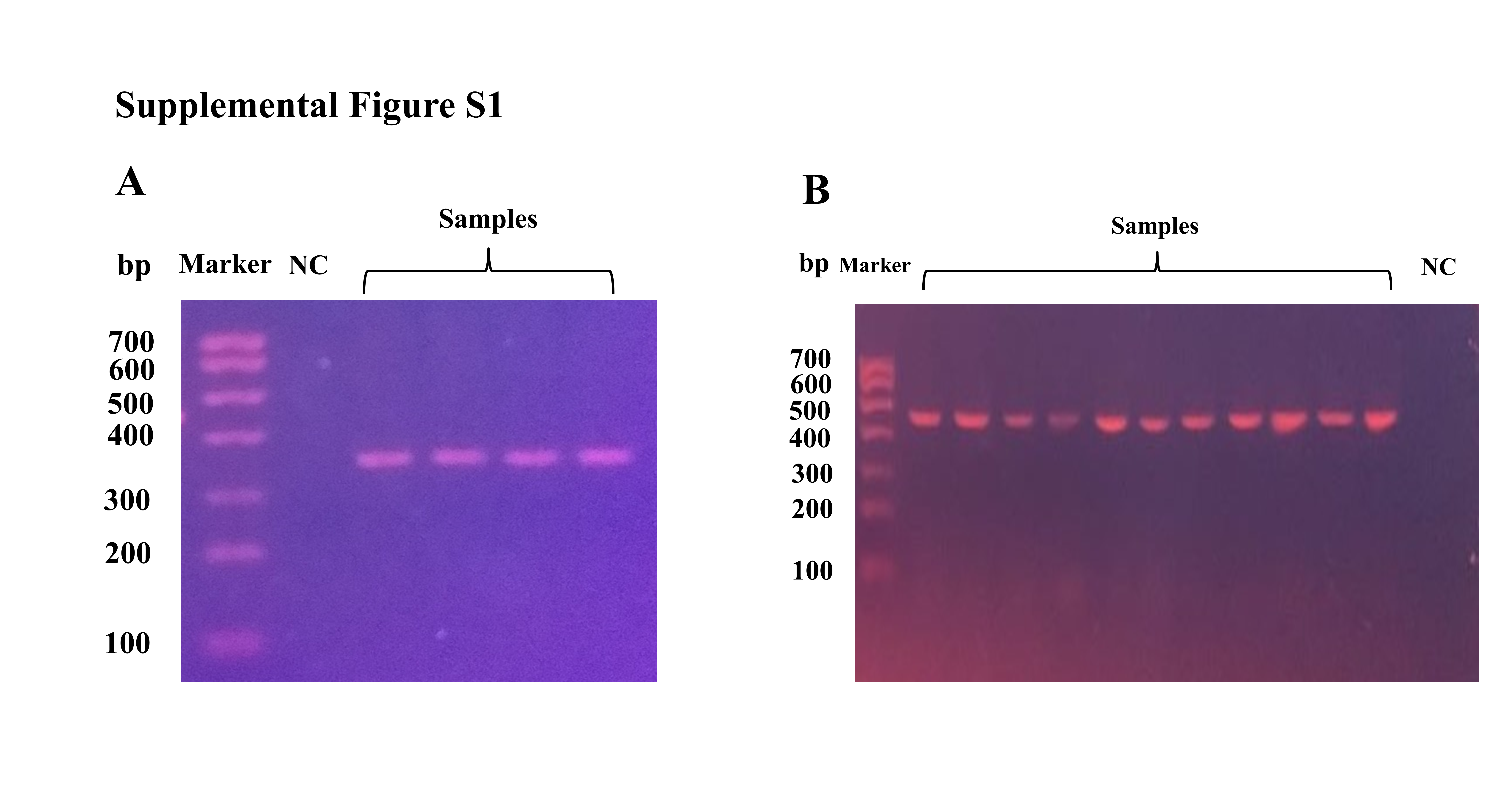

Supplement: Supplementary Figure 1 — PCR identification of mouse genotypes. The specific genes were verified by PCR according to the protocols of Jackson Laboratory. (A) IL-10 mutant (002250) target band was roughly 312bp. (B) DC mutant (019506) target band was roughly 420bp. NC, negative control. [file Image_1.tif]

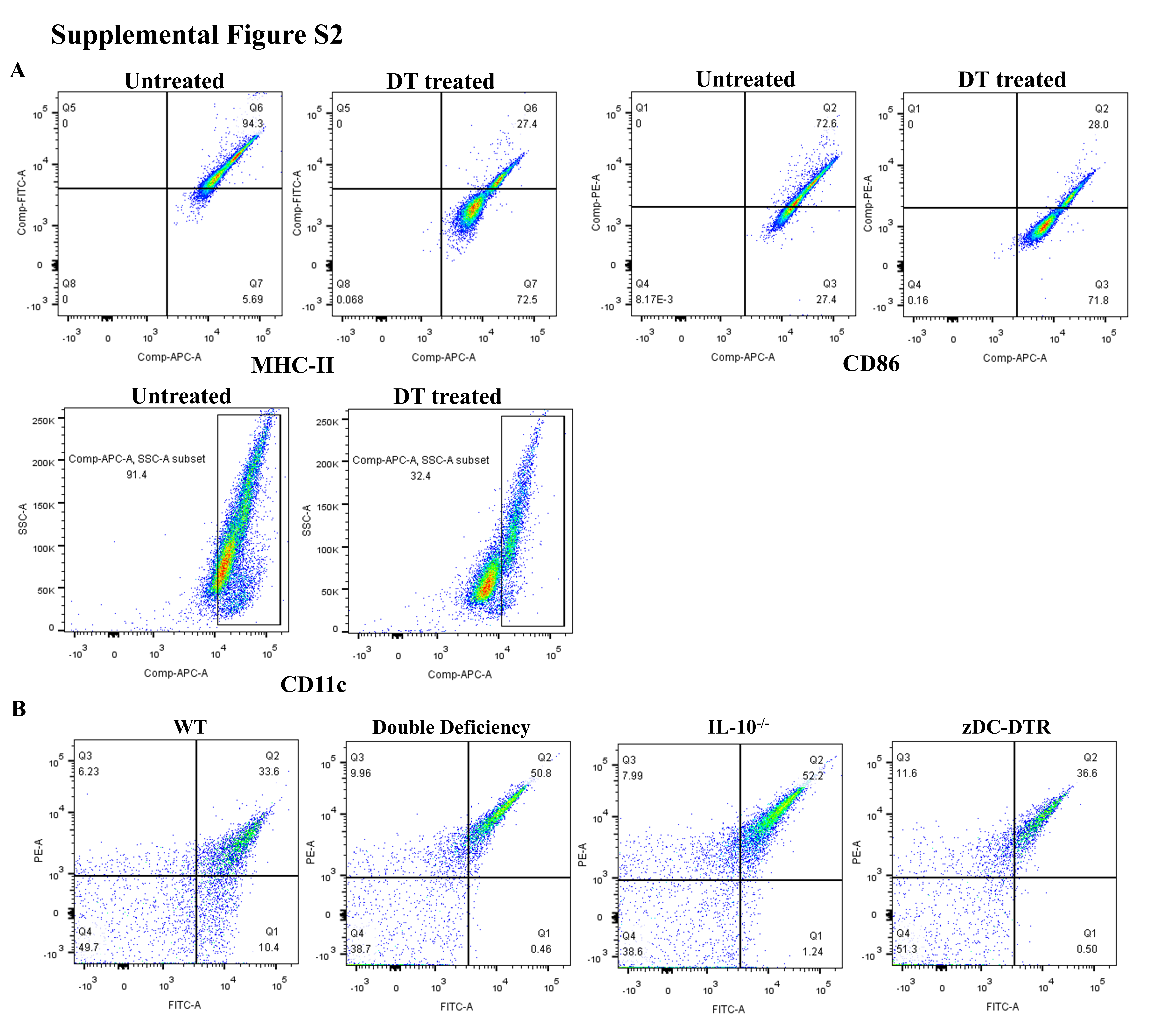

Supplement: Supplementary Figure 2 — DCs deletion efficiency, CD4+ and CD8+ T cells infiltration in the lungs. (A) Mice were euthanized after DT treatment; lungs were collected and digested to a single cell. DCs were labeled with the markers of CD11c-APC, CD86-PE, and MHC-II-FITC to verify deletion of lung residential DCs. CD86 and MHC-II were analyzed by pre-gated CD11c+ cells, CD11c was gated by isotype. (B) Mice were euthanized at 7 dpi and lungs were collected and digested to prepare a single cell. CD4-FITC and CD8-PE were labeled to determine the infiltration in the lungs. CD4 and CD8 were analyzed by pre-gated CD3+ cells. [file Image_2.tif]

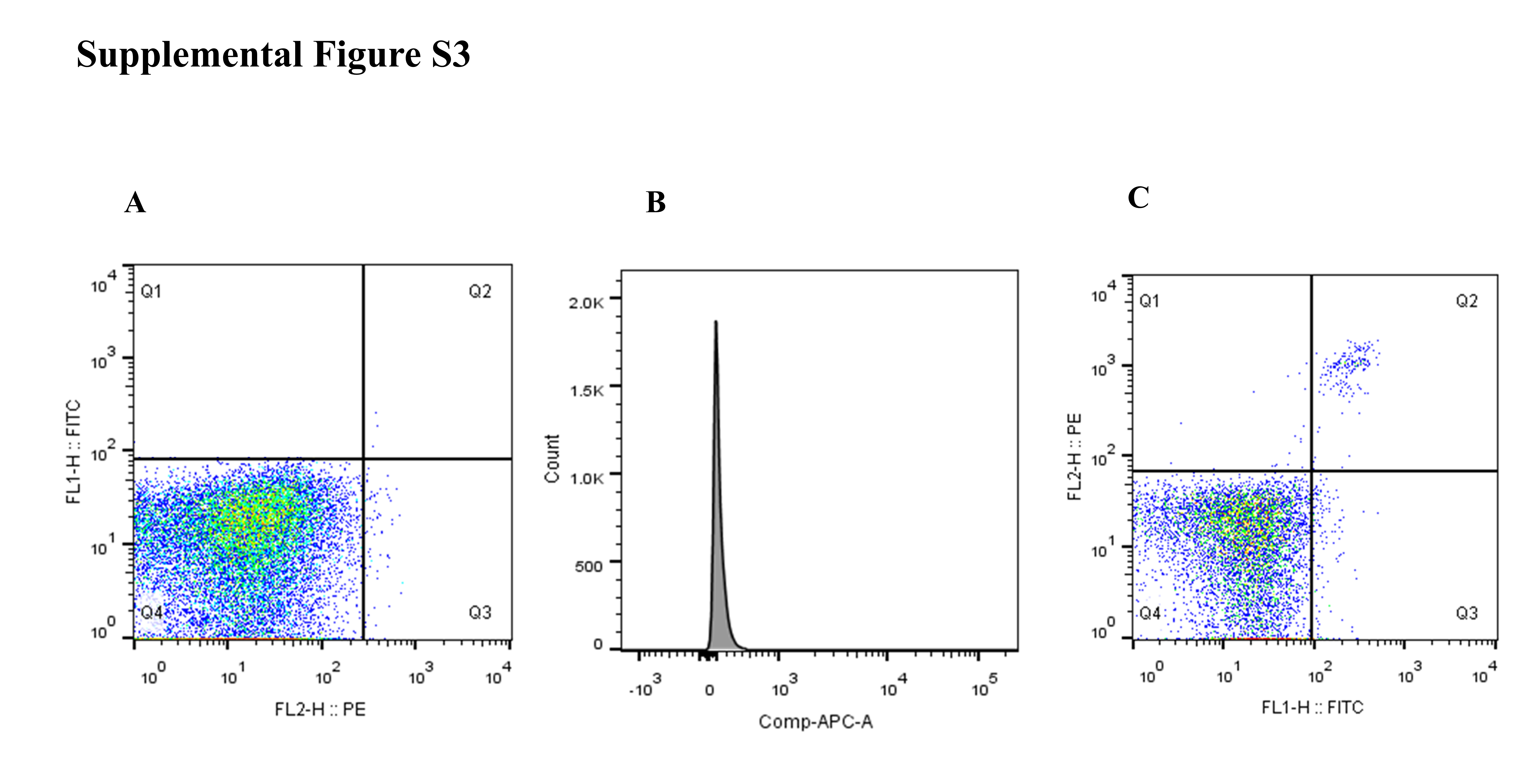

Supplement: Supplementary Figure 3 — The isotype control of flow cytometry. (A) Detecting isotype of CD4+ and CD8+ T lymphocyte (CD4-FITC, CD8-PE). (B) Detecting IDO isotype (IDO-APC). (C) The negative control of apoptosis (Annexin V-FITC, PI-PE). Flow cytometry refers to isotype to set gates for further analysis. [file Image_3.tif]

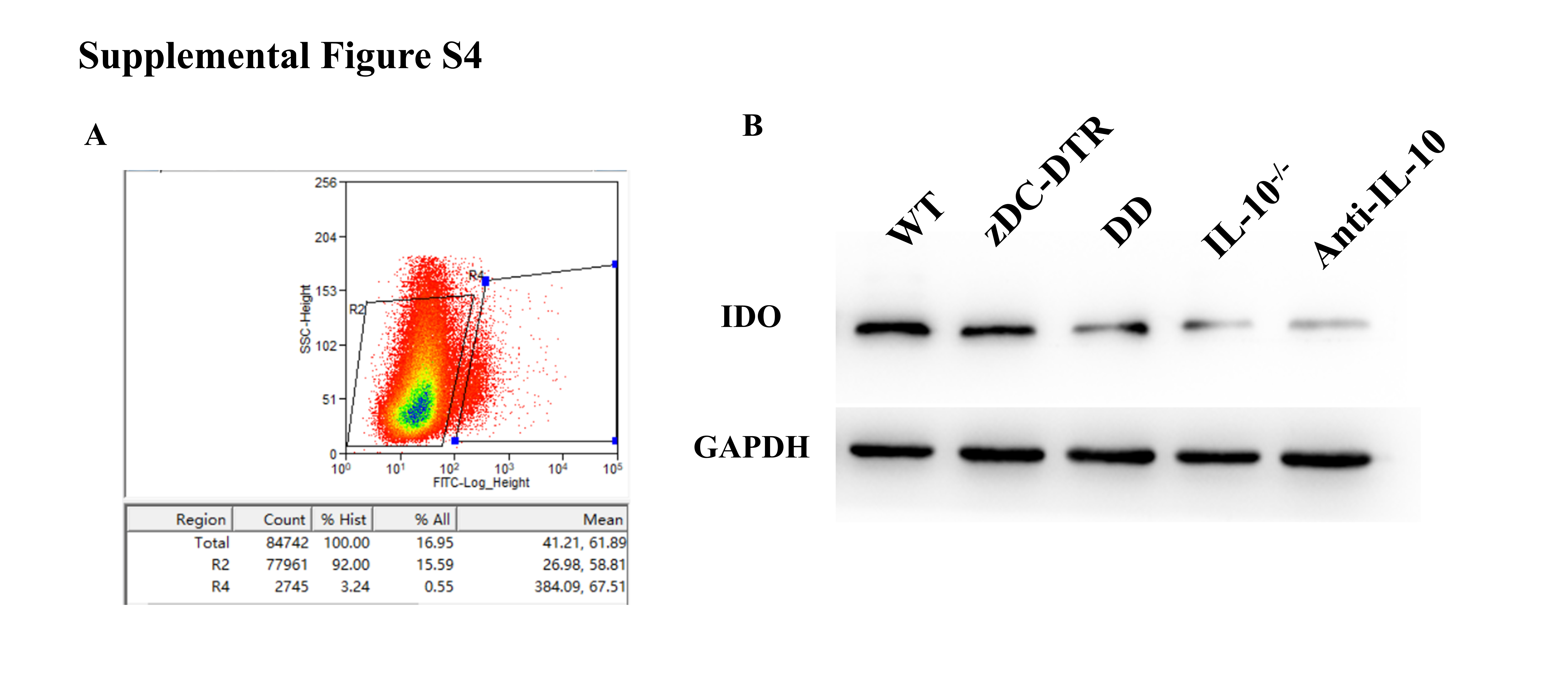

Supplement: Supplementary Figure 4 — IDO expression in the lungs by Western blotting. (A) Lung residential DCs were isolated by BD FACSMelody™ Cell Sorter. The proportion of DCs was roughly 3% of lung cells. (B) Western blotting detection of IDO expression in the lung at 72 hpi. IDO expression increased significantly in DCs from the zDC-DTR mice and WT mice (*P<0.01). On contrary, IDO expression was decreased obviously in the DD mice, the IL-10−/− mice, and the anti-IL-10 mice. [file Image_4.tif]

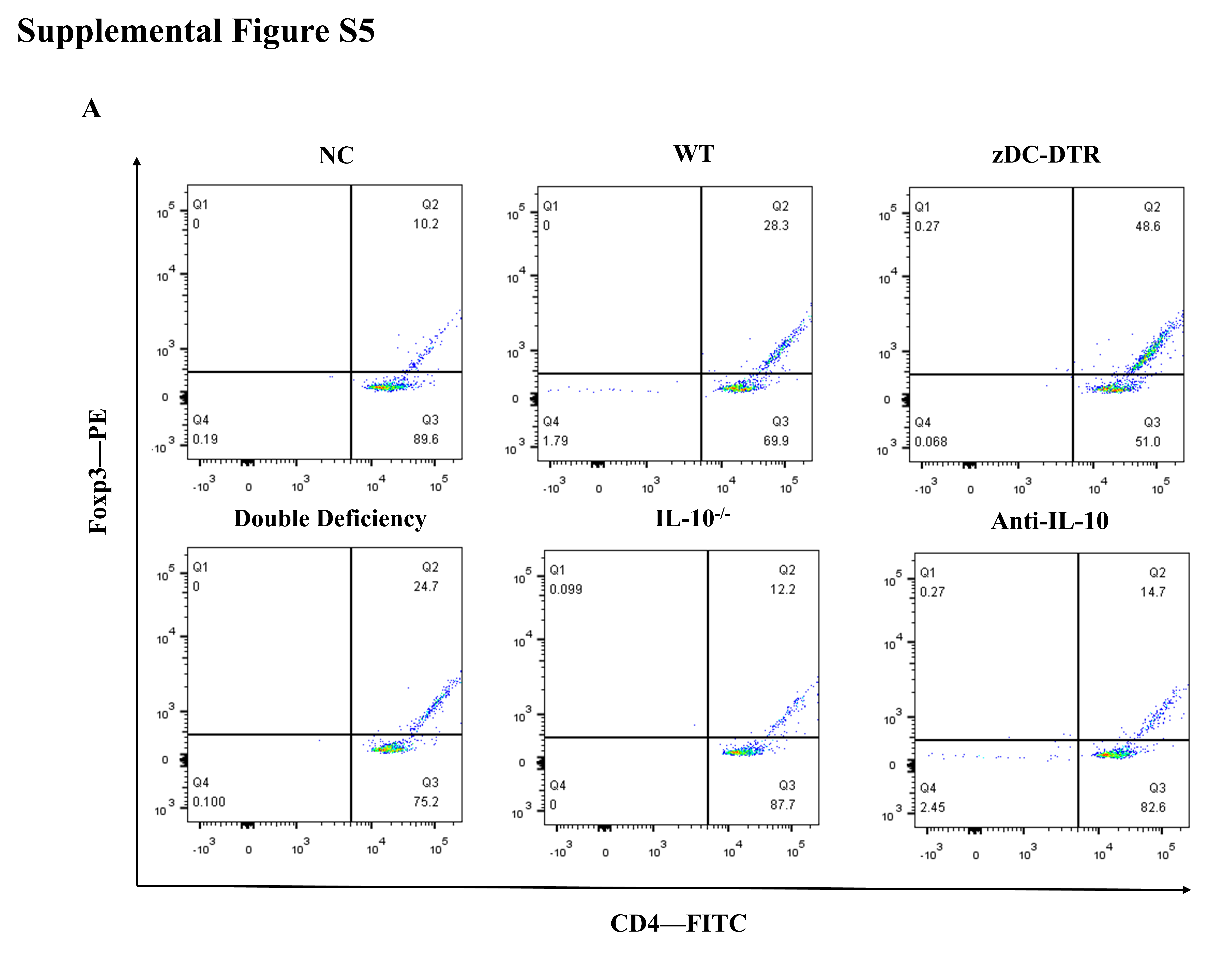

Supplement: Supplementary Figure 5 — Detection of Foxp3 expression by flow cytometry. (A) Cells were collected and stained with surface markers (CD4), then fixed and permeabilized. This was followed by incubation with a nuclear antibody (Foxp3). Finally, cells were stained with fluorescent secondary antibodies. Goat anti-rabbit IgG-PE was used as the secondary antibody at 1/2000 dilution. Foxp3 expression was significantly decreased in the IL-10-/- group and Anti-IL-10 group compared to the zDC-DTR group. A similar trend was comparable to the expression by Western blotting. [file Image_5.tif]

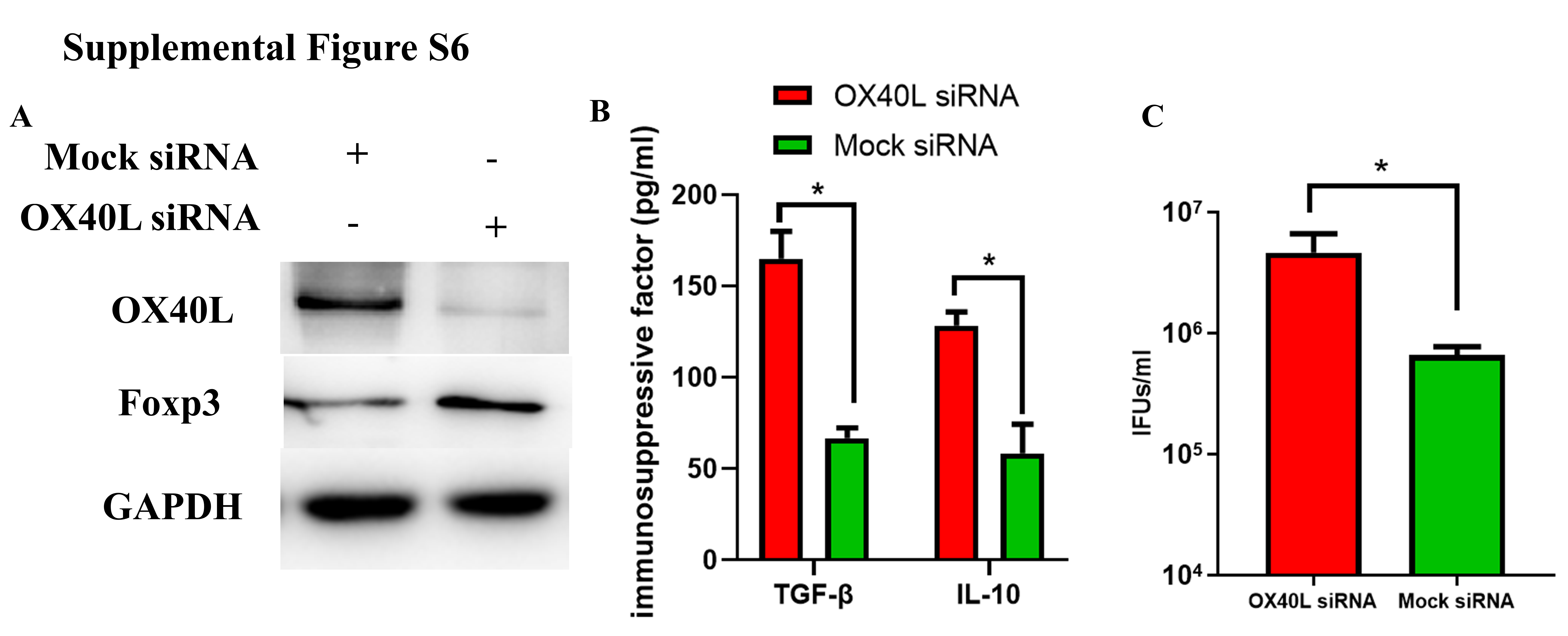

Supplement: Supplementary Figure 6 — siRNA blocked the OX40-OX40L pathway. (A) Co-culture system was treated OX40L siRNA and mock siRNA in 24-well plate, respectively. After 24h treatment, cells were infected at an MOI of C. psittaci. At 72 hpi, cells were collected and assayed for the expression of OX40L and Foxp3 by Western blotting. The expression of Foxp3 significantly increased after OX40L siRNA treatment. (B) TGF-β and IL-10 were collected and determined by ELISA kits in the co-culture system supernatants. Higher expression of the TGF-β and IL-10 were found in the OX40L blocked group. (C) After 24h siRNA treatment, cells were infected at an MOI of C. psittaci. Post 72h infection, cells were collected and lysed by ultra-sonication, infectious progeny EBs were counted using IFU assay. All data were expressed as the mean ± SD (n=5 per group). The differences were analyzed by ANOVA (*P<0.05). [file Image_6.tif]
